# Supplementary material for: Tumour Necrosis Factor-α, Chemokines, and Leukocyte Infiltrate Are Biomarkers for Pathology in the Brains of Venezuelan Equine Encephalitis (VEEV)-Infected Mice
Source: Viruses. 2023 May 31;15(6):1307. doi: 10.3390/v15061307 (PMC10302690; doi:10.3390/v15061307)
Supplement: Supplementary file 1 [file viruses-15-01307-s001.zip › viruses-2386231-supplementary.pdf]

Supplemental figures

Supplemental table 1: The trajectories of the multiple measurements taken from Balb/c mice infected with either $23\ pfu$ (red) or $4140\ pfu$ (black) VEEV TrD via the subcutaneous route. Animals were culled at pre-determined time points post infection. Each data point is taken from a different animal and the line is the geomean of the group.

Analyte	Brain	Blood	Spleen
**TNF-α**<br>**Method: Luminex**	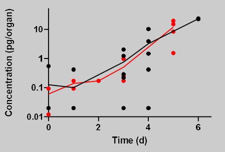	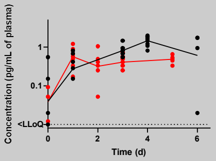	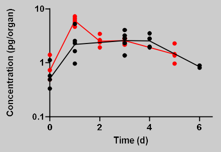
**CCL2 (MCP-1)**<br>**Method: Luminex**	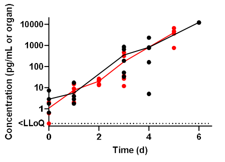	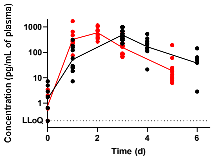	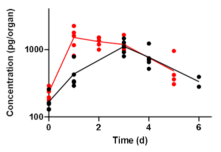
**CCL-5 (RANTES)**<br>**Method: Luminex**	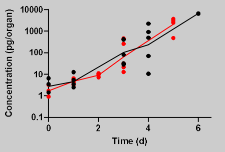	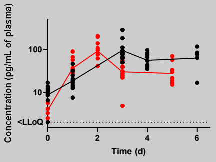	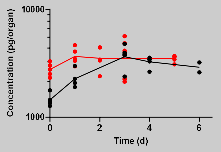
**CD45+ cell count**<br>**Method: Flow cytometry**	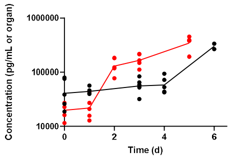	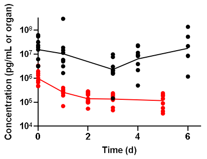	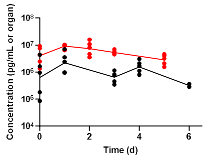
**IL-6**<br>**Method: Luminex**	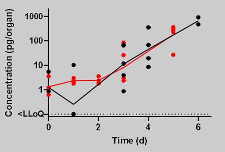	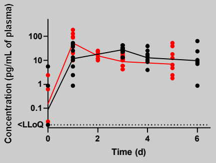	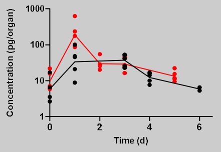
**IL-1α**<br>**Method: Luminex**	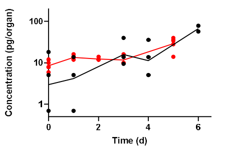	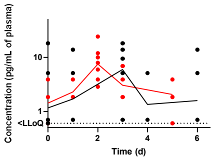	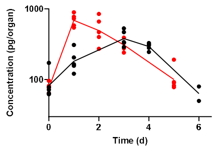
**CXCL-1 (KC)**<br>**Method: Luminex**	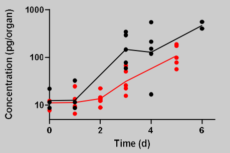	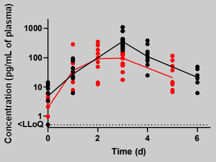	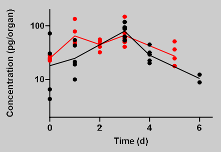
**MMP-9**<br>**Method: Luminex**	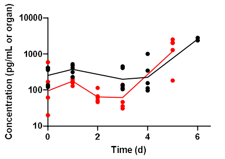	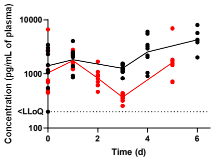	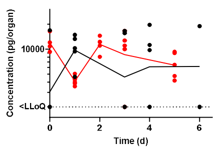
**Viral count**<br>**Method: Plaque assay**	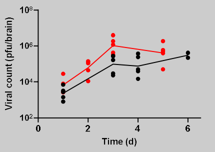	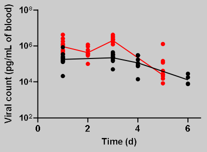	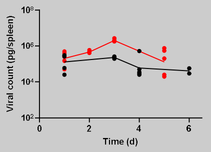

Analyte	Brain	Blood	Spleen
**IFN-γ**<br>**Method: Luminex**	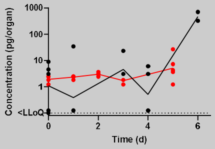	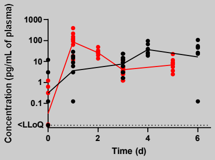	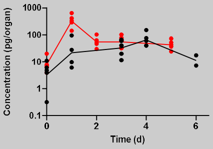
**IL-1β**<br>**Method: Luminex**	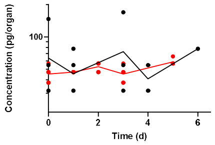	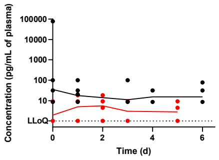	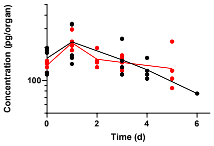
**IL-10**<br>**Method: Luminex**	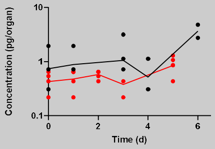	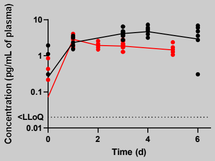	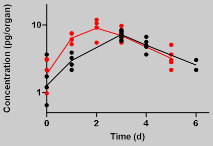
**β-NGF**<br>**Method: Luminex**	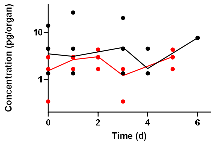	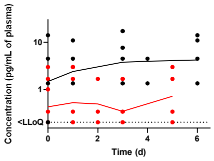	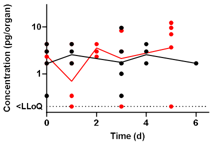
**IL-17**<br>**Method: Luminex**	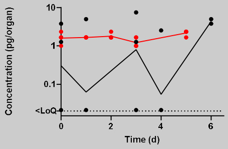	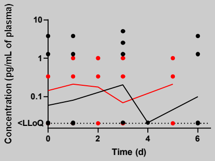	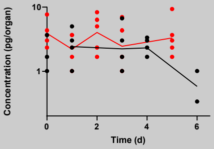
**IL-4**<br>**Method: Luminex**	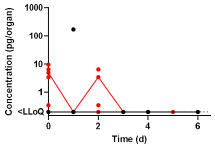	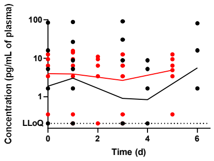	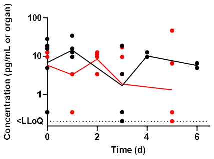
**Organ weight**<br>**Method: scales**	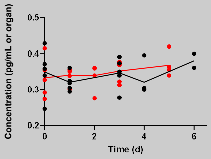	Not applicable	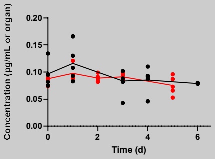
**Ly6G+ cell count**<br>**Method: Flow cytometry**	Not taken	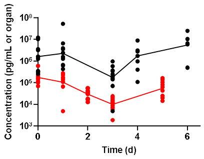	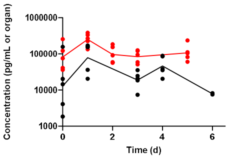
**CD11b+ cell count**<br>**Method: Flow cytometry**	Not taken	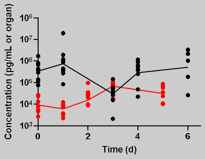	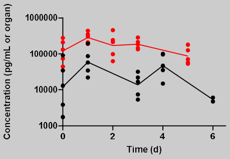
**CD3+ cell count**<br>**Method: Flow cytometry**	Not taken	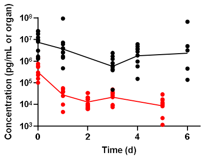	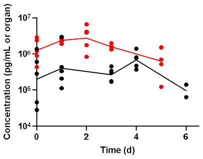

Analyte	Brain	Blood	Spleen
**CD19+ cell count**<br>**Method: Flow cytometry**	Not taken	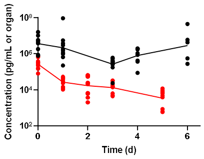	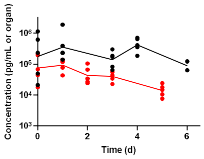
**Ly6G+ cell proportion**<br>**Method: Flow cytometry**	Not taken	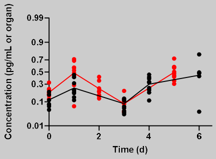	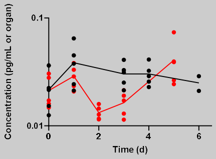
**CD11b+ cell proportion**<br>**Method: Flow cytometry**	Not taken	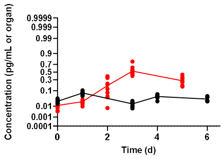	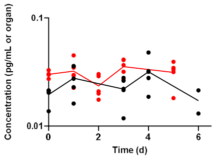
**CD3+ cell proportion**<br>**Method: Flow cytometry**	Not taken	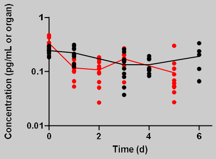	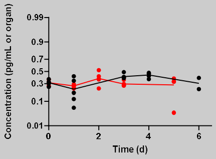
**CD19+ cell proportion**<br>**Method: Flow cytometry**	Not taken	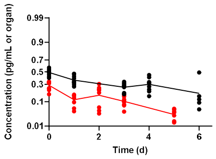	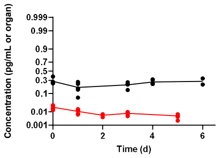

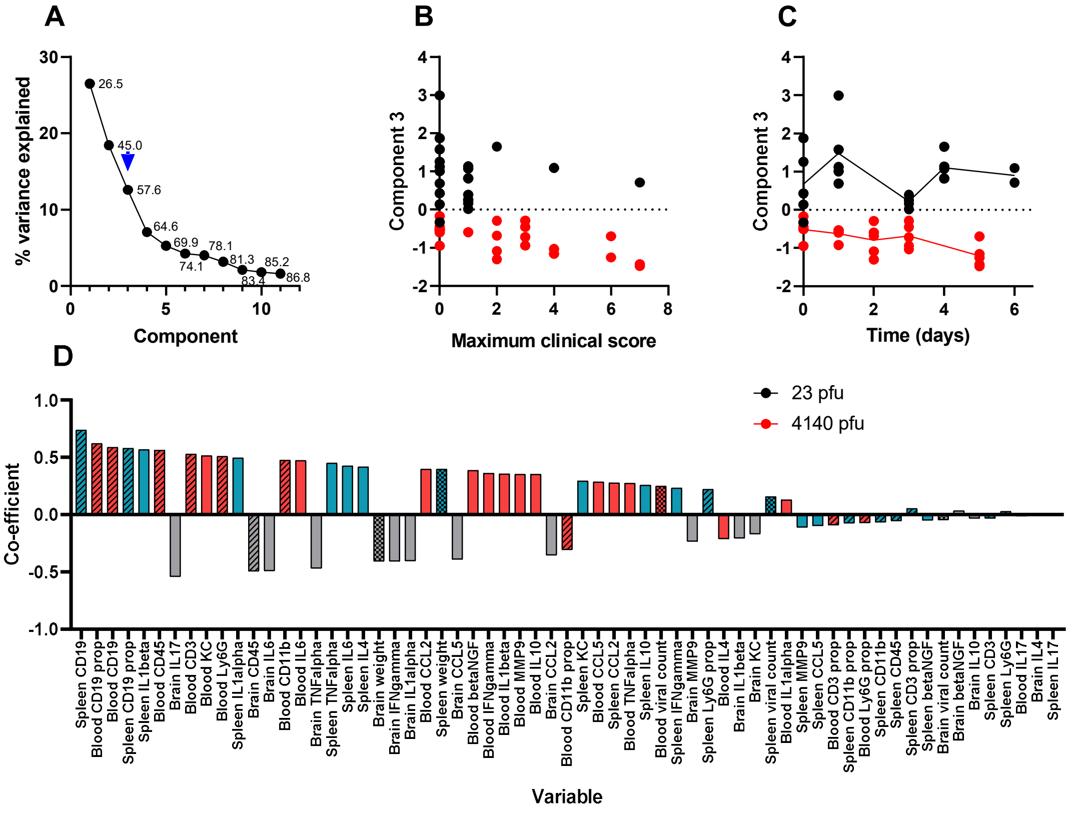

Supplemental figure 1: The third component in a PCA of time course data from Balb/c mice infected with $(23\ pfu)$ or $(4140\ pfu)$ VEEV TrD via the subcutaneous route. Animals were culled at time points and multiple measurements taken. <u>Panel A</u> shows the scree plot indicating the proportion of the dataset (including the data from all parameters) that can be described by each component (an amalgam of some of each parameter) calculated by the analysis. This data is shown either as the line (the proportion explained by the component) or as a data label (the cumulative proportion). An arrow has been added to indicate which component is characterised further in this figure (i.e. component 3 in this figure). <u>Panel B</u> show the regression-derived value for this component, for each mouse, relative to the maximum clinical score prior to cull. Each data point is from a single mouse. <u>Panel C</u> show the regression-derived value for this component, for each mouse, relative to time post challenge at point of cull. Each data point is from a single mouse with a line added to indicate the mean for each cull point. <u>Panel D</u> shows the coefficients, in order of absolute scale, of each variable that contribute to this component. Measurements of viral titre/body weight, cytokines and flow cytometry are individually assigned in blood (red), spleen (blue) and brain (grey). These measurements are further divided by viral titre and animal weight (checkerboard), flow cytometry (crosshatched) and cytokine (plain).

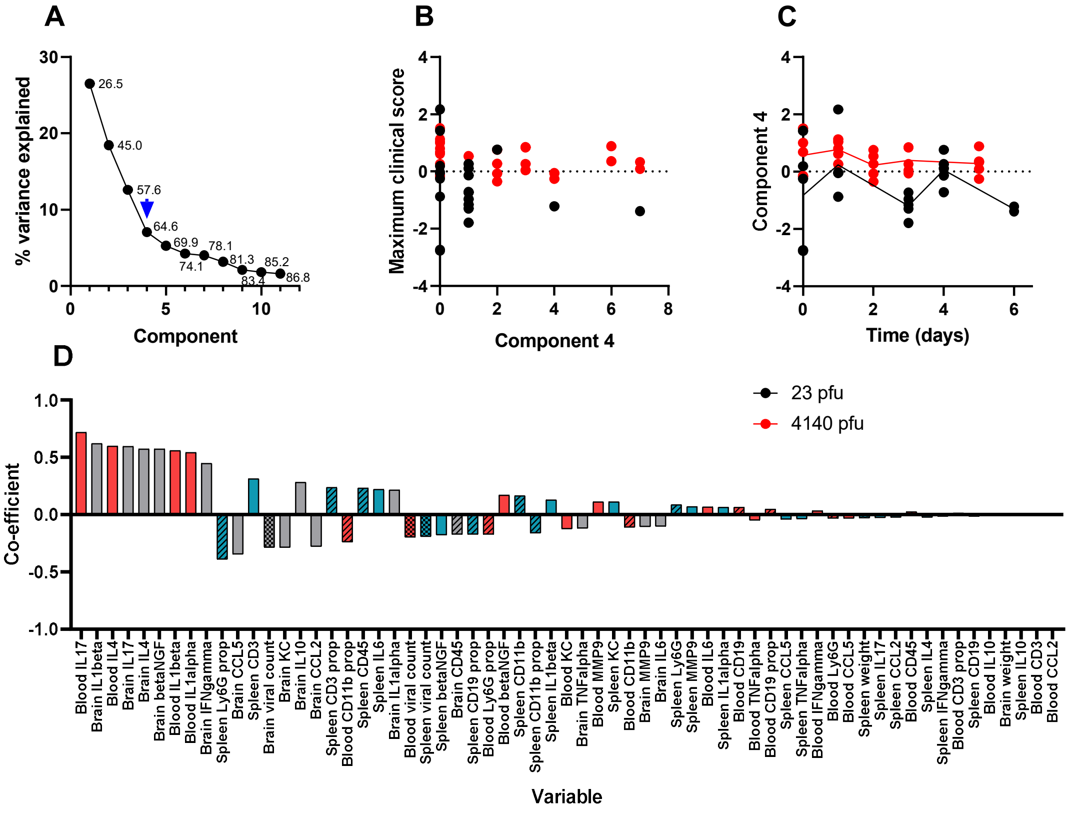

Supplemental figure 2: The forth component in a PCA of time course data from Balb/c mice infected with $(23\ pfu)$ *or* $(4140\ pfu)$ *VEEV TrD via the subcutaneous route. Animals were culled at time points and multiple measurements taken.* *Panel A* *shows the scree plot indicating the proportion of the dataset (including the data from all parameters) that can be described by each component (an amalgam of some of each parameter) calculated by the analysis. This data is shown either as the line (the proportion explained by the component) or as a data label (the cumulative proportion). An arrow has been added to indicate which component is characterised further in this figure (i.e. component 4 in this figure).* *Panel B* *show the regression-derived value for this component, for each mouse, relative to the maximum clinical score prior to cull. Each data point is from a single mouse.* *Panel C* *show the regression-derived value for this component, for each mouse, relative to time post challenge at point of cull. Each data point is from a single mouse with a line added to indicate the mean for each cull point.* *Panel D* *shows the coefficients, in order of absolute scale, of each variable that contribute to this component. Measurements of viral titre/body weight, cytokines and flow cytometry are individually assigned in blood (red), spleen (blue) and brain (grey). These measurements are further divided by viral titre and animal weight (checkerboard), flow cytometry (crosshatched) and cytokine (plain).*

The data file with data in raw unanalysed from:

VEEV complete dataset.csv
